# Evaluation of Continuous Membrane Chromatography Concepts with an Enhanced Process Simulation Approach

**DOI:** 10.3390/antib7010013

**Published:** 2018-03-02

**Authors:** Steffen Zobel-Roos, Dominik Stein, Jochen Strube

**Affiliations:** Institute for Separation and Process Technology, Clausthal University of Technology, Leibnizstraße 15, D-38678 Clausthal-Zellerfeld, Germany; zobel-roos@itv.tu-clausthal.de (S.Z.-R.); dominik.stein@sartorius-stedim.com (D.S.)

**Keywords:** continuous chromatography, membrane chromatography, process modeling, continuous bio-manufacturing

## Abstract

Modern biopharmaceutical products strive for small-scale, low-cost production. Continuous chromatography has shown to be a promising technology because it assures high-capacity utilization, purity and yield increases, and lower facility footprint. Membrane chromatography is a fully disposable low-cost alternative to bead-based chromatography with minor drawbacks in terms of capacity. Hence, continuous membrane chromatography should have a high potential. The evaluation of continuous processes goes often along with process modeling. Only few experiments with small feed demand need to be conducted to estimate the model parameters. Afterwards, a variety of different process setups and working points can be analyzed in a very short time, making the approach very efficient. Since the available modeling approaches for membrane chromatography modules did not fit the used design, a new modeling approach is shown. This combines the general rate model with an advanced fluid dynamic distribution. Model parameter determination and model validation were done with industrial cell cultures containing Immunoglobulin G (IgG). The validated model was used to evaluate the feasibility of the integrated Counter Current Chromatography (iCCC) concept and the sequential chromatography concept for membrane adsorber modules, starting with a laboratory-type module used for sample preparation. A case study representing a fed-batch reactor with a capacity from 20 to 2000 L was performed. Compared to batch runs, a 71% higher capacity, 48.5% higher productivity, and 38% lower eluent consumption could be achieved.

## 1. Introduction

Biopharmaceutical production is undergoing a paradigm shift. The number of blockbuster products is decreasing [1,2,3]. Stratified medicine is a promising approach for future pharmaceutical products. Mixtures of active components and their dosages are tailored for higher effectiveness and less side effects [4]. However, this goes along with lower sales volumes, thus higher specific production costs.

One approach to significantly lower production costs is continuous manufacturing [5,6]. In the upstream processing, this is done mainly with perfusion technology [7,8,9], whereas, in the downstream processing, the idea is to simply switch the well-known platform process. This goes along with continuous chromatography [10,11]. Although not very common for monoclonal antibodies, continuous chromatography is widely used in pharmaceutical production [12]. For most bioseparations, however, the classical simulated moving bed is not suitable [13,14]. Thus, a lot of different chromatographic modes were invented. Sequential chromatography, like SMCC (Sequential Multicolumn Chromatography) [15], PCC (Periodic Counter Current Chromatography) [9,16,17], CaptureSMB (Simulated Moving Bed) [18,19], and BioSMB [20,21], seems the preferred method at the moment. 

Simply converting the well-known platform process, however, misses out on the potential of non-chromatographic or not classical chromatographic unit operations. Aqueous two-phase liquid–liquid extraction, for example, is a fully continuous process with low costs and complexity but high potential for monoclonal antibodies [22,23,24,25]. The same is true for precipitation [26,27].

Besides different unit operations, there is a huge potential for chromatographic separations that are not particle-based, such as membrane- or fiber-based chromatography [28,29,30,31,32,33,34].

This potential should be evaluated. This can be done efficiently with process simulation. After building and validating a batch membrane adsorption model, this can be used to simulate all types of chromatographic setups. This approach is rather fast and cheap [35,36].

**Figure 1:**
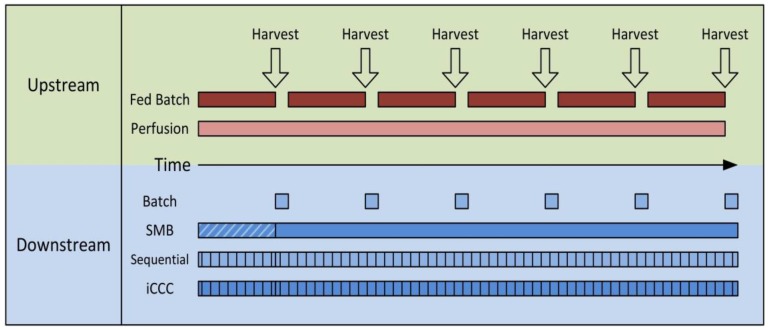
Schematic overview of the scheduling differences in upstream and downstream processing for batch and continuous production. In the upstream Fed Batch and Perfusion, in the Downstream Bachth; SMB, Simulated Moving Bed; Sequential; iCCC, integrated Counter Current Chromatography.

As shown in Figure 1, the task of continuous chromatography processes is the continuous purification of either a discontinuous feed stream from a batch or fed-batch reactor or a continuous feed stream from a perfusion reactor. A complete continuous operation, meaning fully continuous feed and product stream, is hard to obtain with most “continuous” chromatography processes in bioseparations. Simulated Moving Bed (SMB), Multicolumn Countercurrent Solvent Gradient Purification (MCSGP) in a six-column setup [37,38], and BioSMB [20,21] are fully continuous. The latter, however, is often used with continuous feed loading only. Simply put, most of the other semi-continuous processes can be seen as a chain of many batch operations. These are often somehow linked and run in cycles leading to a cyclic steady state. In the case of a batch or fed-batch upstream, these processes run the whole time between two harvests, reducing the column size and facility footprint significantly. Of course, a stable product is needed that does not have to be processed in minimum time. For perfusion reactions, a low but steady feed stream is produced, making large columns inefficient. 

In both cases, the total loading capacity needed in the process is reduced significantly. Also, the number of cycles introduces a new degree of freedom. In a given timeframe, a few runs with high-capacity columns can process the same amount of feed as fast-operated columns with lower capacity. This is very much in favor of membrane chromatography, since membrane adsorber modules can be operated very fast.

Of all the continuous or semi-continuous processes mentioned before, sequential chromatography and integrated Counter Current Chromatography (iCCC) were tested.

The classical three- or four-zone simulated moving bed chromatography does not seem to be suitable for membrane chromatography in bioseparations, since it can only perform a binary separation [39,40], and only one step gradient at the feed inlet is possible [14]. Although Multicolumn Counter Current Solvent Gradient Purification (MCSGP), precisely twin-column MCSGP, has proven to work well with classical chromatography columns [41,42], it is neglected in favor of iCCC.

**Figure 2:**
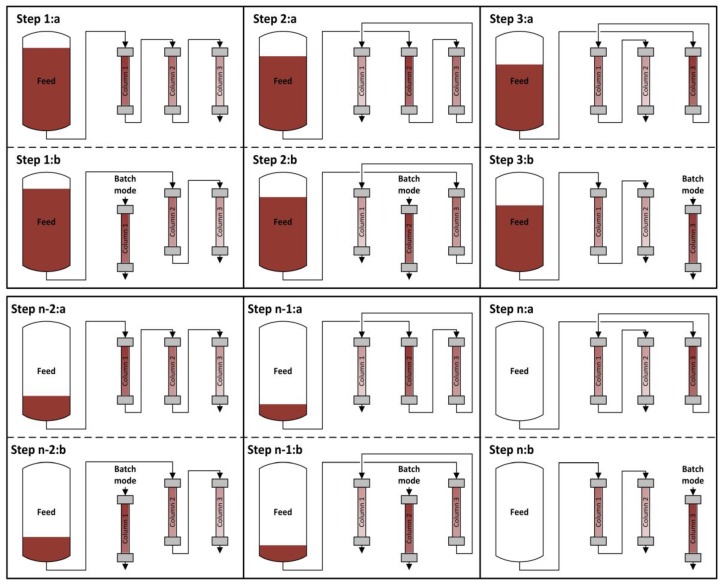
Scheduling of loading and separation phases for a three-column sequential chromatography after batch upstream.

The principal approach for sequential chromatography is shown in Figure 2. The process sequence can generally be separated in as many steps as there are columns. Angarita et al. used two protein A columns for continuous capture of monoclonal antibodies [18]. Mahajan et al. did the same with three columns [17], and Holzer et al. with four columns [15]. Warikoo et al. used a four-hydrophobic-interaction column setup for the capture of enzymes [9].

Each step can be separated further into the loading phase (a) and the batch phase (b). In the loading phase, the columns are linked together. Unlike batch chromatography, the first column of the chain is not loaded to a certain low percentage of breakthrough, since the breakthrough is not going to waste but is loaded onto the following columns. After loading is completed, the column is decoupled and runs through washing, elution, and regeneration steps, very similar to the classical batch operation.

The amount of feed loaded on the column and the number of columns used is a matter of optimization and is mostly affected by the loading speed and the time needed for the batch operation step.

**Figure 3:**
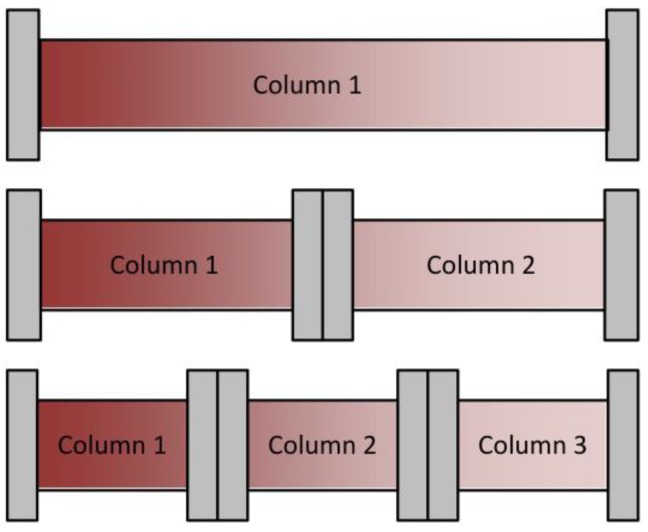
Schematic overview of column capacity utilization for different numbers of columns in the loading zone.

Besides achieving continuous feed loading, the main benefit of this method is a higher column capacity utilization, as indicated in Figure 3. Warikoo et al. achieved a 50% higher resin capacity utilization [9]. A higher resin capacity utilization usually goes along with higher productivity. Although the comparison between batch and continuous chromatography is complicated because of the different approaches and optimization tasks, Baur et al. found an average productivity increase of 39% at an average capacity increase of 46% [19].

**Figure 4:**
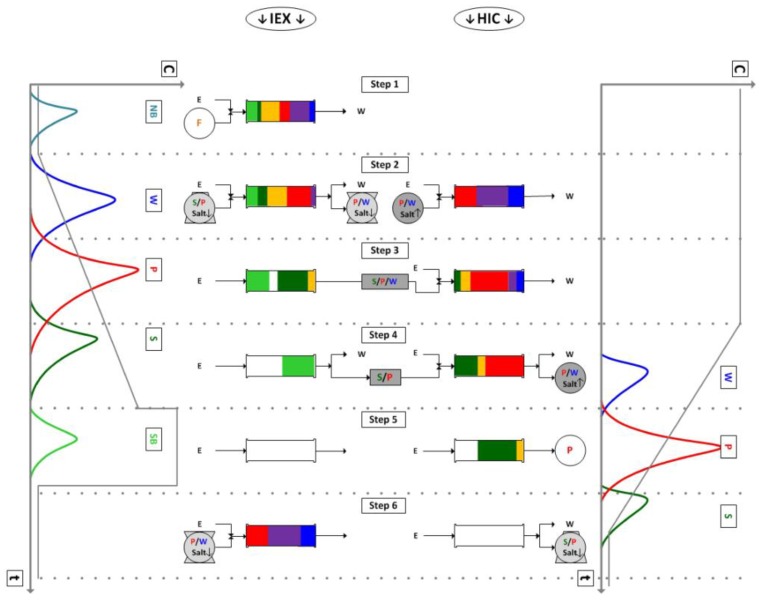
Sequence of an integrated Counter Current Chromatography (iCCC) cycle. IEX, Ion-Exchange Chromatography; HIC, Hydrophobic Interaction Chromatography; NB, Non-Binding; W, Weak-Binding; P, Product; S, Strong-Binding; SB, Stronger-Binding; F, Feed.

The second semi-continuous chromatography step investigated for its use with membrane chromatography is the iCCC. It combines two columns or modules with different resin types. These must be operable with the same buffer system but with opposing gradient behaviors, like ion-exchange and hydrophobic interaction resins [43]. One complete iCCC cycle is shown in Figure 4. The main idea is to fractionate the overlapping region of product and side-component peaks and reload it in the next cycle. The overlap of the weak binder and product from the ion-exchange column and the overlap of the strong binder and product from the hydrophobic interaction column elute at the low salt concentrations. This is illustrated in Figure 4, Step 2 and 6. Hence, these get reloaded on the ion-exchange column in the next cycle. The overlap of the product and strong binder on the Ion-Exchange Chromatography (IEX) (Step 4) and of the weak binder and product on the Hydrophobic Interaction Chromatography (HIC) (Step 4) elute with a high salt concentration and are therefore reloaded on the HIC column. This strategy avoids the classical purity/yield cutoff. The iCCC allows for high purity and high yield. Furthermore, the product concentration is increased. A thorough derivation and explanation can be found here [13,44].

The aim of this manuscript is the evaluation of both processes, sequential chromatography and iCCC, for their use with membrane chromatography modules.

## 2. Materials and Methods

### 2.1. Proteins, Buffers, and Columns

All experiments were carried out in 20 mM NaPi Buffer at pH 6.0. For ion-exchange separations, this buffer was used as equilibration buffer A. For elution buffer B, 1 M ammonium sulfate was added. For the hydrophobic interaction membranes, the nomenclature was changed, thus, buffer A contained 1 M ammonium sulfate and buffer B does not.

The membrane chromatography devices were Sartobind^®^ S and Sartobind^®^ Phenyl Nano modules with 8 mm bed height (Sartorius Stedim Biotech Gmbh, Göttingen, Germany), which are typical laboratory-type setups for quick sample preparation. 

For analytical size-exclusion chromatography (SEC), a buffer containing 100 mM sodium sulfate and 100 mM NaPi was used at pH 6.6. All salts were obtained from Merck KGaA, Darmstadt, Germany.

Analytical SEC columns Yarra^®^ SEC-3000 (3 μm, 300 × 4.6 mm) were obtained from Phenomenex Inc., Torrance, CA, USA.

Protein A chromatography was performed with PA ID Poros^®^ Protein A Sensor Cartridges (Applied Biosystems, Waltham, MA, USA).

Immunoglobulins (IgGs) were obtained from a cell culture of an industrial Chinese Hamster Ovary (CHO) cell line and clarified by centrifugation at 1500× *g*.

### 2.2. Devices and Instruments

The experimental setup consisted of a standard VWR-Hitachi LaChrom Elite^®^ HPLC system (VWR international, Darmstadt, Germany) with a quaternary gradient pump L-2130, Autosampler L-2200, and diode array detector L-2455.

### 2.3. Parameter Determination

#### 2.3.1. Fluid Dynamics

The experimental parameter determination of the voidage *ε* with respect to the molecular weight and the determination of the axial dispersion coefficient D_ax_ were done with tracer experiments under non-binding conditions. As tracer molecules, dextran and pullulan were used. Analytical dextran standards were used with a molecular weight of 1 kDa, 5 kDa, 12 kDa, 25 kDa, 50 kDa, 80 kDa, 150 kDa, 270 kDa, and 670 kDa (Fluka/Sigma-Aldrich, St. Louis, MO, USA). Analytical pullulan standards were used with a molecular weight of 342 Da, 1080 Da, 6.1 kDa, 9.6 kDa, 21.1 kDa, 47.1 kDa, 107 kDa, 194 kDa, 337 kDa, and 708 kDa (PSS-Polymer Standards Service GmbH, Mainz, Germany). These where dissolved in buffer A or B at a concentration of 5 g/L. The volumetric flow $\dot{V}$ was 0.5 mL/min, 1 mL/min, 5 mL/min, and 15 mL/min.

The voidage *ε* can be calculated with Equation (1):

(1) $$\varepsilon \left(M\right)=\frac{\left(\bar{t}-\bar{t_{i}}\right)\cdot \dot{V}}{V_{m}}$$

with $\bar{t}$ as the total mean residence time, $\bar{t_{i}}$ as the mean residence time of the instruments and devices without the module, and $V_{m}$ as the volume of the module. The axial dispersion coefficient can be calculated with Equations (2) and (3) [45]:

(2) $$\frac{\sigma^{2}}{{\bar{t}}^{2}}=2\cdot \left(\frac{D_{ax}}{v\cdot l}\right)-2\cdot {\left(\frac{D_{ax}}{v\cdot l}\right)}^{2}\cdot \left[1-e^{-\frac{v\cdot l}{D_{ax}}}\right]$$

(3) $$\frac{\sigma^{2}}{{\bar{t}}^{2}}=2\cdot \left(\frac{D_{ax}}{v\cdot l}\right)+8\cdot {\left(\frac{D_{ax}}{v\cdot l}\right)}^{2}$$

with $\bar{t}$ as the mean residence time, *σ²* as variance, v as flow velocity, and l as the characteristic length. These equations vary in the Danckwerts boundary conditions [46]. Equation (2) assumes closed vessel, whereas Equation (3) assumes open vessel conditions. For particle-based chromatography, the differences in the resulting dispersion coefficient are usually very low.

#### 2.3.2. Isotherms

The static binding capacity was measured with batch adsorption measurements in a way very similar to the one described by Schwellenbach et al. [28]. For the simulation of chromatography in bind and elute mode, the isotherms have to be measured with respect to the modifier concentration. For Sartobind^®^ S, isotherms with 0.02 M, 0.05 M, 0.1 M, 0.15 M, 0.2 M, and 0.3 M ammonium sulfate were measured. For Sartobind^®^ Phenyl, modifier concentrations of 0.2 M, 0.5 M, 0.6 M, 0.8 M, and 1 M ammonium sulfate were investigated. To adjust the salt concentration, diafiltration was done using a Sartoflow^®^ Slice 200 benchtop system with Sartocon^®^ Slice 200 Hydrosart^®^ cassettes (Cutoff 2 kDa, Sartorius Stedim Biotech Gmbh, Göttingen, Germany). 100 mL cell free culture was diafiltrated with a 10 fold buffer exchange for each salt concentration. The IgG concentration after diafiltration was around 5 g/L, with roughly 1 g/L of total side component concentration.

For each isotherm, five data points with different IgG concentrations were measured. Amounts of 1 mL, 2 mL, 3 mL, 5 mL, and 6 mL of feed were mixed with the buffer of the corresponding salt concentration to achieve a total volume of 6 mL. From this, 1 mL was taken, and the IgG and side component concentrations were measured by protein A and size-exclusion chromatography.

A round, 13 mm single layer membrane was added to the remaining 5 mL and agitated for 16 h with 120 rpm. After that, a 1 mL sample of the supernatant was taken and analyzed by protein A and size-exclusion chromatography.

The membrane was extracted, transferred to 5 mL of buffer with the initial salt concentration, and agitated for 2 h.

Finally, the membrane was placed in 5 mL elution buffer containing 1 M ammonium sulfate for the ion-exchange membrane module and no ammonium sulfate for the hydrophobic interaction module. Anyways, the HI buffer was not salt-free since it still contained salts from the sodium phosphate buffer. Thus, it was marked as 0 M for not containing ammonium sulfate. The elution sample was agitated for 2 h. Both wash and elution supernatants were analyzed by protein A and size-exclusion chromatography.

The mass balances of the binding and of the elution steps both give the membrane capacity *q_i_*. Equation (4) applies to the binding, while Equation (5) to the loading step:

(4) $$q_{i}=\frac{\left(c_{Feed,i}-c^{*}{}_{i}\right)\cdot \;V_{total}}{V_{Ads}}$$

(5) $$q_{i}=\frac{c^{*}{}_{i}\cdot \;V_{total}}{V_{Ads}}$$

where *c_Feed,i_* is the concentration of component *i* before shaking, and c^*^_i_ is the concentration after shaking; c^*^_i_ is not the same in both equations, since the equations apply to two different steps (binding and elution).

To implement the equilibrium into the process model, a Langmuir type isotherm [47] with an additional linear term was used:

(6) $$q_{i}=\frac{H_{i}\cdot c_{i}}{1+\sum_{j=1}^{n}\frac{H_{j}}{q_{max,j}}\cdot c_{j}}+L_{1}\cdot c_{mod}\cdot c_{i}$$

*H_i_* is the Henry coefficient of component *i*, *q_max_* is the maximum loading capacity. The linear coefficient *L*_1_ is only needed for the hydrophobic interaction membrane. To account for the salt dependency, the Henry coefficient and the maximum loading were implemented as functions of the modifier concentration *c_mod_*:

(7) $$q_{max,i}=a_{1,i}\cdot c_{mod}+a_{2,i}$$

(8) $$H_{i}=b_{1,i}\cdot c_{mod}{}^{b_{2,i}}$$

The experimental data points were plotted against the theoretical data points calculated with Equation (6). The parameters *a*_1,*i*_, *a*_2,*i*_, *b*_1,*i*_, and *b*_2,*i*_ were then determined by minimizing the least-squares error between the measured and the calculated data points.

#### 2.3.3. Model Validation

For the validation of the fluid dynamic, the experiments from Section 2.3.1 were used. For the validation of the whole model, gradient separations were done. For each module, three chromatographic runs with different gradient steepness were done. The gradients had a length of 1 Column Volume (CV), 3 CV, and 5 CV at a volumetric flow of 0.5 mL/min. Prior to the gradient, a 2 CV wash step took place. The injection volume was 99 μL with 1 g/L IgG titer and a total of 0.2 g/L side component concentration, which is typical for the cell culture chosen as a first feasibility study [48,49,50].

## 3. Process Modeling

Process modeling is commonly known to work well for bead-based chromatography [47,51,52], especially for the process design of continuous processes [39,53,54,55,56]. For membrane chromatography, a lot of research on process modelling has also been done [29,57,58,59,60].

### 3.1. Fluid Dynamics, Mass Transport, and Isotherms

Because of the similarity between bead-based and membrane chromatography, the membrane itself was implemented as a general rate model [61,62,63]. The membrane in the membrane chromatography module is wrapped around an inner cylinder. The inlet and outlet as well as the membrane housing are flown through in axial direction. The membrane itself has a radial flow. This leads to a more complicated fluid dynamic for such a membrane module compared to column chromatography.

**Figure 5:**
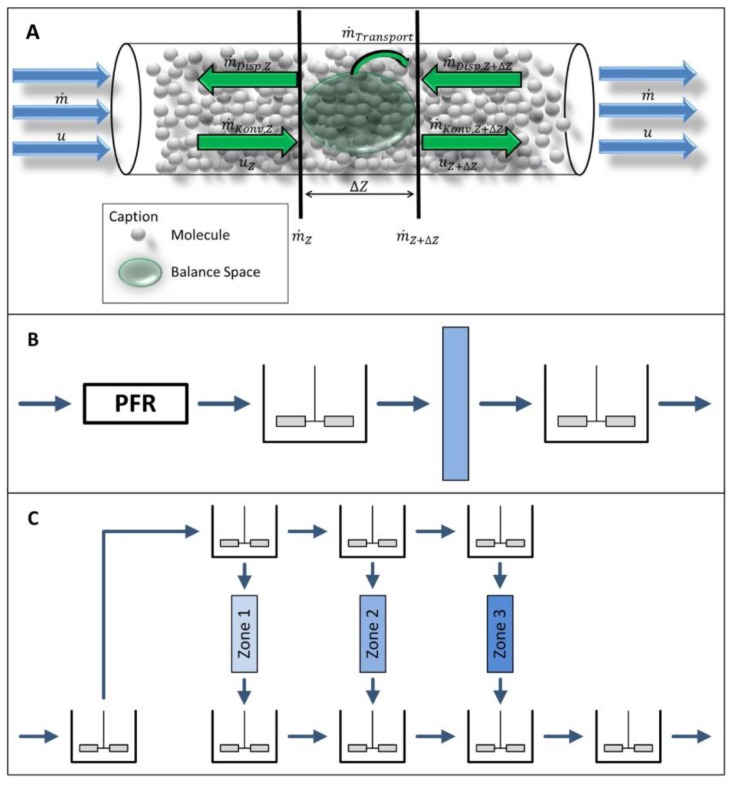
Overview of different fluid dynamic models. (**A**) Distributed plug flow model; (**B**) Roper–Lightfoot model [64]; (**C**) zonal rate model [59,60]. PFR, Plug Flow Reactor.

This aspect was already addressed by Roper et al. [64] who implemented stirred tanks before and after the column, as shown in Figure 5B. A further improvement is the multi-stage approach called “zonal rate model”, first introduced by Francis et al. (Figure 5C) [59,60,65,66].

**Figure 6:**
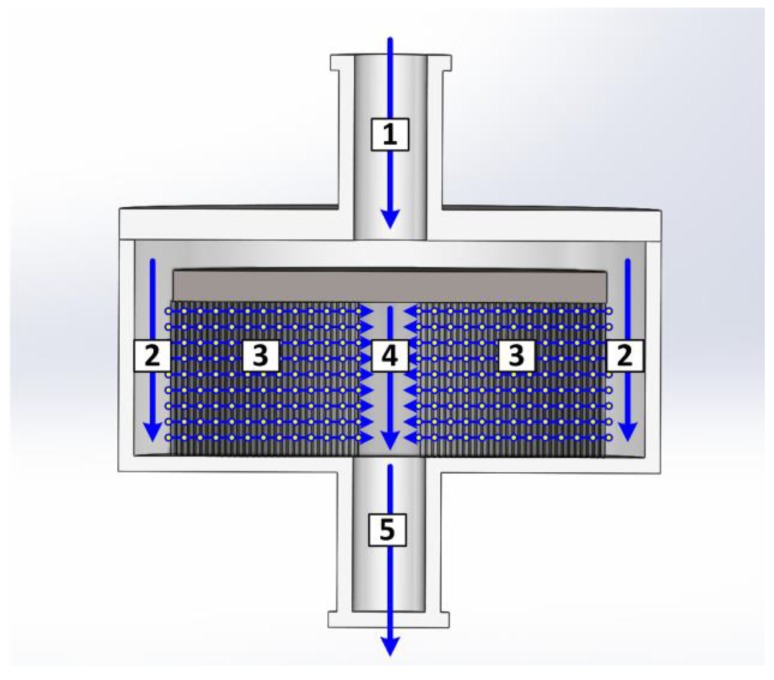
Fluid dynamic distribution in a membrane chromatography module.

In this work, a different approach was chosen. The module was subdivided into five areas as shown in Figure 6. These were the inlet (1) and outlet (5), the annular gap (2), the inner cylinder (4), and the membrane itself (3). Each zone was described by an individual mass balance.

Please note that, for better clarity, indices indicating the flow direction, *x* for axial and *r* for radial, were left out as long as the balance room of the equation only had one direction. 

Zone 1 was implemented as a plug flow pipe:

(9) $$\frac{\partial c_{i}}{\partial t}=-u\cdot \frac{\partial c_{i}}{\partial x}+D_{ax}\cdot \frac{\partial^{2}c_{i}}{\partial x^{2}}$$

with *u* as the linear velocity and *D_ax_* as the axial dispersion coefficient.

Zone 2 was also implemented with Equation (9). Additionally, for each discrete counterpart, a secondary mass balance was implemented that split the volumetric flow. One part entered the membrane and the rest went further along zone 2. It is assumed that the fluid dynamic resistance was constant alongside the membrane surface and that the concentration of all three streams was identical, reducing the mass to a fluid balance:

(10) $${\dot{V}}_{x}={\dot{V}}_{x+1}+{\dot{V}}_{r}$$

Here, the indices *x* and *r* represent the axial and the radial direction, respectively.

Zone 3 represented the membrane:

(11) $$\frac{\partial c_{i}}{\partial t}=-u_{int}\cdot \frac{\partial c_{i}}{\partial r}+D_{ax}\cdot \frac{\partial^{2}c_{i}}{\partial r^{2}}-kla\left(c_{i}-q_{i}\right)$$

*u_int_* is the interstitial velocity, which takes porosity into account. The mass transfer zone was simplified by the factor *kla.* This combines the mass transfer resistance coefficient and the effective mass transfer area, since both are relatively hard to determine or measure. The loading *q_i_* was implemented with Langmuir type isotherms, as mentioned earlier (Equations (6) to (8)).

Zone 4 was mainly described by Equation (9). Similar to Zone 2, a second mass balance was needed, this time to combine the streams leaving the membrane:

(12) $${\dot{V}}_{x+1}\cdot \;c_{i,x+1}\;={\dot{V}}_{x}\cdot \;c_{i,x}+{\dot{V}}_{r}\cdot \;c_{i,r}$$

Here, the concentrations were not necessarily the same.

Zone 5 was a copy of zone 1.

### 3.2. Model Validation

The model parameter determination showed a total voidage *ε* of 0.8 and no dependency from the molecular weight of the tracer, as expected for membrane chromatography.

The axial dispersion coefficient determined with Equations (2) or (3) proved to be only slightly helpful. If the coefficient determined with these equations was applied for each zone equally, the simulations did not match the tracer experiments. Obviously, each zone had its own share to the total fluid dynamics. Thus, the axial dispersion coefficients were obtained by adapting the parameters for each zone. The best results were obtained with 9 × 10^−4^ cm^2^/s for zones 1 and 2, 5 × 10^−3^ cm^2^/s for the membrane (zone 3), and 250 cm^2^/s for zones 4 and 5. The axial dispersion coefficients for Zones 1 to 3 were in a typical order of magnitude for these setups. The Bodenstein numbers for the inlet, which can be calculated with Equation (13) [45], varied between 30 and 120. This indicated plug flow behavior. The critical value for the transition from plug flow to stirred tank was 5, with dispersion dominating at lower Bodenstein numbers [45]. 

(13) $$\mathrm{Bo}=\frac{u\cdot l}{D_{ax}}$$

For the outlet zones 4 and 5, the axial dispersion coefficients were unusually high. The corresponding Bodenstein numbers were 6.6 × 10^−5^ for zone 4 and 1.1 × 10^−4^ for zone 5. These zones acted like ideal stirred tank reactors. Regarding the flow regime (see Figure 6), this seemed reasonable. In zone 4, the axial flow was constantly mixed with a radial flow from every side. This cross flow regime should lead to extensive mixing.

**Figure 7:**
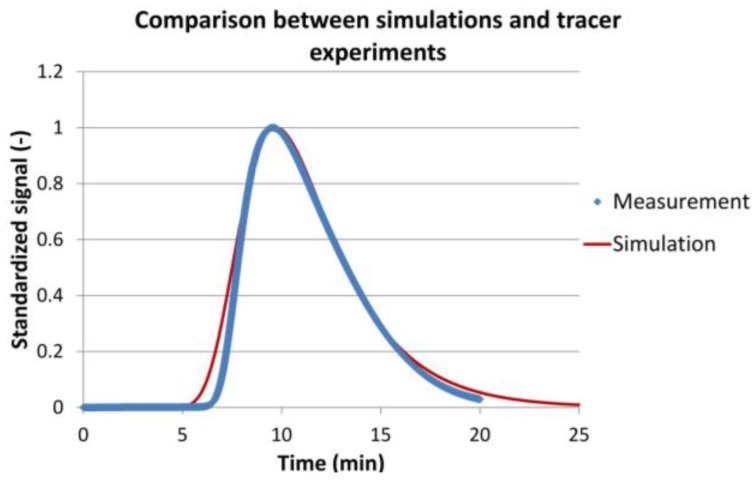
Comparison between simulations (red line) and tracer experiments (blue dots) for a volumetric flow of 0.5 mL/min.

Figure 7 shows an example of the experimental and simulation results for 0.5 mL/min volumetric flow. The experimental results are represented by blue dots. The peak shape shows a rather steep front and a lot of tailing. The simulation results are represented by the red curve. It can be seen that both curves are in good agreement. When comparing the mean residence times of the experiments with the simulations for all volumetric flows, a deviation of less than 0.8% was found. The coefficient of determination reached an average of 0.984 for the different volumetric flows.

**Figure 8:**
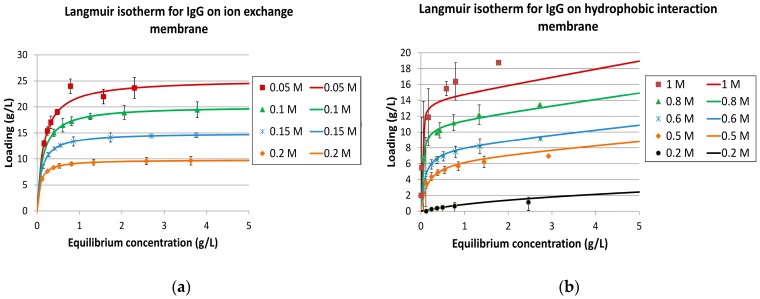
Langmuir isotherms for immunoglobulins (IgG) on ion-exchange membrane (IEX) (**a**) and hydrophobic interaction membrane (HIC) (**b**).

The isotherm experiments were interpreted with IgG as the target component (ID 1) and two key components. Component ID 2 represented the weak binder, while ID 3 was the strong binding component. The salt-dependent IgG isotherms are shown in Figure 8.

It should be noted that the experimental approach is not suitable for a scientifically accurate competitive Langmuir parameter determination. This would need a variation in the concentration ratio of all three components. Furthermore, the side component concentration in this example was relatively low, making IgG a dominant component, which is typical for modern cell culture fermentation. However, for any conceptual process design evaluation, the presented fast-forward approach is sufficient.

**Figure 9:**
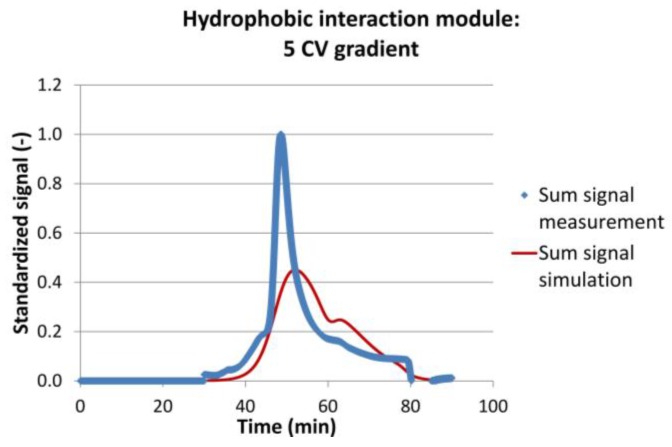
Experimental (blue dots) and simulation (red line) results for the hydrophobic interaction module with a 5 Column Volume (CV) gradient, as an example for simulation results with a low coefficient of determination (*R*² = 0.647).

Table 1 sums up the results of the model validation. It can be seen that the mean residence time of the most important IgG peak was met with good accuracy. The maximum deviation was −7%, the minimum deviation almost 0%, and the mean deviation about 2.5%. The coefficient of determination was not as good as for the fluid dynamic experiments. It varied between 0.65 and 0.95 with an average value of 0.81. The mass balance for each run was similar, with less than 5% deviation. Figure 9 shows the chromatograms for the data set with the least agreement between experiments and simulations.

It can be seen that the peak start and end were met well. Also, the maximum of the IgG peak and the maximum of the much smaller, strong binder peak were met. The simulated peaks however were more Gaussian-shaped, whereas the experimental peak has a rather sharp front but a long tailing. Again, this example represent the worst result obtained. The divergence might be a result of not sufficiently accurate isotherm parameters, of simplified *kla* assumptions, or of differing fluid dynamics. Nevertheless, for any conceptual process design evaluation of continuous processes, the results are sufficient [36,52,67].

## 4. Results and Discussion

The model validation showed a good agreement between experimental and simulated membrane chromatography runs. In the following, the model will be used for process feasibility studies.

### 4.1. Integrated Counter Current Chromatography (iCCC)

**Figure 10:**
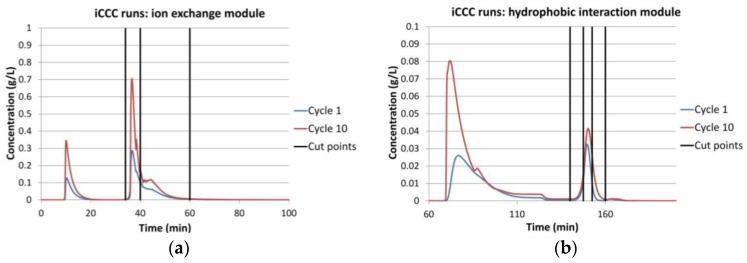
iCCC simulation runs: (**a**) ion-exchange module (**b**) hydrophobic interaction module.

The validated model of one membrane chromatography module can be combined with other modules to simulate various continuous processes. For the integrated Counter Current Chromatography (iCCC), one ion-exchange and one hydrophobic interaction module were combined. A simulation run for this setup is shown in Figure 10.

For the given modules, no reasonable operation point could be found. This was mainly due to the large tailing. Figure 10a shows the chromatogram of the ion-exchange module. It can be seen that it has a sharp front and a long tailing. The vertical lines show the fractionation cut point. The fraction between the first and the second line was reloaded on the same module in the following cycle. The fraction between line two and three was loaded on the hydrophobic interaction module. Because of the sharp front, the first fraction could be set to be either very small, which makes it unnecessary, or to contain most of the IgG. The latter case is shown in this example. This was not sensible either, since only little product was transferred to the next column. Hence, it accumulated in the ion-exchange column, quickly outmatching its capacity.

Because of the cut point choice, only little product quantity was transferred to the hydrophobic interaction module (Figure 10b). This led to a small peak and therefore to small fractions. The first fraction was loaded on the HIC module in the following cycle. The second fraction was the final product fraction. The third fraction was loaded on the IEX module. The recycled fractions (1 and 3) did not contain much product, and this hindered a concentration increase in this module.

All things considered, iCCC is not advisable for such membrane chromatography modules, which are designed for laboratory sample preparation as well as scale up devices for large scale flow through applications in industrial manufacturing of biomolecules, with the given component system.

### 4.2. Sequential Chromatography

The feasibility of sequential chromatography was tested with ion-exchange modules. Feed loading was done with a volumetric flow of 3 CV/min. Washing, elution, regeneration, and re-equilibration was done with 5 CV/min.

**Figure 11:**
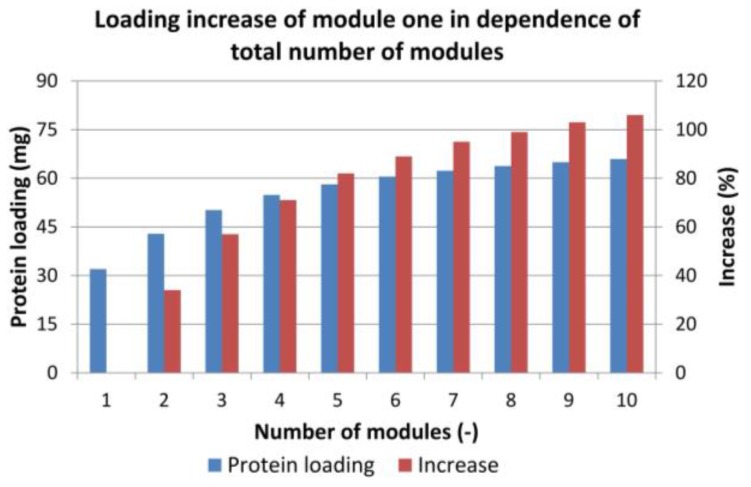
Increase in total loading of the first adsorber module depending on the number of modules used in the sequential loading step. The blue columns indicate the protein loading of the first column, the red columns represent the loading increase compared to only one module.

To estimate the ideal number of modules in line (feed loading, Figure 2 step a), at first, the increase in protein loading was tested. The first module in the chain was loaded until the breakthrough in the last column occurred. Figure 11 shows the amount of protein loaded on the first module (blue columns) and the increase in protein loading (red columns) compared to a single batch run. As expected, the total amount of protein that could be loaded on the first column increased with the number of columns but with decreasing tendency. The increase from one to two modules was 34%, and, after seven columns, the increase raised only by 4% or less.

**Figure 12:**
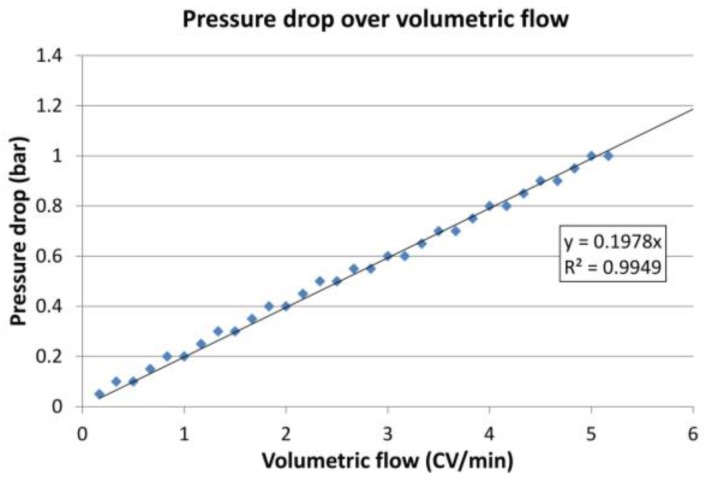
Experimental determination of the pressure drop of one module depending on the volumetric flow.

The ideal number of modules does not depend only on the protein loading. Each module increases the total pressure drop. The maximum pressure in the first module, however, must not exceed 4 bar. Hence, the volumetric flow in the loading zone had to be decreased with increasing numbers of modules. The correlation between the volumetric flow and the pressure drop of one module is shown in Figure 12.

The ideal setup depends on the application. In the following case studies, a batch downstream process and a sequential process were compared. Both shared the same upstream, which was a fed batch reaction with a fermenter volume of 20 L, 200 L, or 2000 L. The IgG titer was 5 g/L. The total amount of batches per year was 40, resulting in a maximum of 4 kg, 40 kg, or 400 kg of product per year if no yield loss occurred [5,11,68].

All processes shared the same elution profile after feed loading, starting with 3 CV wash step, 6 CV gradient, 3 CV regeneration, and 3 CV equilibration. These steps were done with 5 CV/min volumetric flow. As mentioned before, the flow of the loading step in the batch process was 5 CV/min also. For the sequential, continuous processes, four modules with a feed flow of 3 CV/min were found as the ideal combination. This resulted in 2.4 bar backpressure from the modules themselves. Combined with the backpressure of the devices and instruments, a total of 3.5 bar pressure for module one was reached.

With four modules in line, the capacity increased by 71%. This increase outperformed the lower feed flow, resulting in higher productivities. These were 48.5% higher for the continuous compared to the batch run, calculated on the basis of the duration of each cycle/run. Using the same membrane module for batch or continuous operation, the continuous process had 32.7% less processing time. Despite batch chromatography, continuous chromatography has an additional degree of freedom. This is the total process runtime. Referring to Figure 1, one might want to process the reactor volume as fast as possible and use the chromatography site for other tasks while waiting for the next harvest. Another possibility is that the site runs continuously for a longer period, even from one harvest to the following one. This allows for much smaller columns or modules and reduces plant downtime significantly. In this case, the productivity should be calculated from harvest to harvest, giving the continuous process a 71% higher productivity compared to the batch run.

Furthermore, the necessary module size for the continuous process can be reduced significantly. The 20 L and 200 L harvest volumes can easily be processed with 150 mL instead of 5 L modules, a factor about 30 times lower. If a stable production is presumed, a 20 L harvest could be processed with 3 mL nanomodules within 7 days between two harvests. This would need a change in the elution profile from 3 CV to 2 CV washing and from 6 CV to 5 CV gradients. All process parameters and results are listed in Table 2.

Besides a higher productivity, the continuous process had a 38% lower eluent consumption. Since the actual separation process was almost the same for the batch and the sequential case, no significant change in purity or yield was found.

## 5. Conclusions

A new fluid dynamic approach to membrane chromatography modeling was introduced. Both fluid dynamic and total model validation showed that the new approach worked well for the given membrane adsorber module, starting with a laboratory-scale type applied originally for sample preparation. Nevertheless, the parameter determination of the axial dispersion coefficient should be enhanced by analyzing the single module parts instead of the whole module at once. The approach will now be applied to other modules.

**Figure 13:**
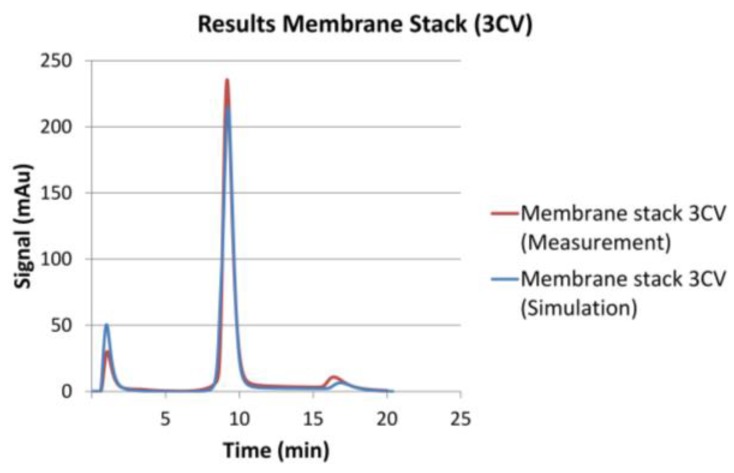
Simulation and experimental results for a membrane stack in a glass column.

The continuous chromatography concept analysis showed that iCCC did not work well for this membrane type, built for quick laboratory sample purification and this set of components. This was mainly due to a considerable peak tailing. This did not result from the membrane itself. A cross-check experiment with 30 round, 1 cm membrane layers in a Superformance^®^ 300-10 (Götec-Labortechnik GmbH, Bickenbach, Germany) showed absolute symmetric peaks, as shown in Figure 13. The chromatographic setup (IgG test system, NaPi buffer etc.) was exactly the same as for the rest of this paper. The simulations were done with a classical General Rate Model as for bead-based chromatography without a complicated fluid dynamic distribution.

Sequential chromatography could be shown to work well for membrane chromatography modules. Compared to batch runs, a 71% higher capacity, at least 48.5% higher productivity, and 38% lower eluent consumption could be achieved. The downstream processing could be done in either 32.7% less time or with much smaller membrane modules.

The case study assumed fed-batch reactors. The trend in upstream processing, however, shows preference for continuous perfusion reaction. These usually run at a perfusion rate between one and three, which results in a relatively low feed flow rate. Here, even very small membrane chromatography modules in a sequential process should give enough capacity at very low costs and are fully disposable.

Module design for processing, however, should be different from laboratory sample purification, with minimized tailing [29]. 

## Figures and Tables

**Table 1:** Overview of the simulation results for IgG on ion-exchange and hydrophobic interaction membranes.

Module	Volumetric Flow	Gradient	Mean Residence Time (IgG)			Coefficient of Determination
			Experiment	Simulation	Deviation	*R*²
	(mL/min)	(CV)	(min)	(min)	(%)	(-)
Ion exchange	0.5	1	38.73	38.09	1.66	0.864
		3	47.20	47.18	0.04	0.777
		5	53.60	52.28	2.46	0.770
Hydrophobic interaction	0.5	1	40.26	39.42	2.09	0.945
		3	46.54	47.18	−1.38	0.848
		5	53.51	57.36	−7.19	0.647
	Mean value				2.468	0.809

CV, Column Volume.

**Table 2:** Process parameters and results for the 20 L, 200 L, and 2000 L fermenter case studies.

		20 L Fermenter			200 L Fermenter			2000 L Fermenter	
		Batch	Sequential		Batch	Sequential		Batch	Sequential
Total membrane volume	(L)	15	9		150	88		1500	877
Module size	(L)	5	0.15	0.003	5	5	0.15	5	5
Number of modules per cycle	(-)	1	4	4	1	4	4	1	4
Runs/cycles	(-)	3	15	731	30	4	4	300	44
Runtime	(h)	0.16	3.66	183.24	1.63	1.10	36.65	16.33	10.99
Productivity									
Run/Cycle	(g/L/day)	2939	4366		2939	4366		2939	4366
Batch to batch	(g/L/day)	1.11	1.90		1.11	1.90		1.11	1.90
Eluents consumption	(L/g)	2.45	1.52		2.45	1.52		2.45	1.52

## Abbreviations

c (mg/mL)	Concentration
CV (mL)	Column Volume
D_ax_ (cm^2^/s)	Axial Dispersion Coefficient
H (-)	Henry Coefficient
HIC	Hydrophobic Interaction Chromatography
iCCC	integrated Counter Current Chromatography
IEX	Ion-Exchange Chromatography
IgG	Immunoglobulin G
l (cm)	Length
kla (1/s)	Effective Mass Transfer Resistance and Area
MCSGP	Multicolumn Counter Current Solvent Gradient Purification
NaPi	Sodium Phosphate Buffer
PCC	Periodic Counter Current Chromatography
PFR	Plug Flow Reactor
q (mg/mL)	Loading
SEC	Size-Exclusion Chromatography
SMB	Simulated Moving Bed
SMCC	Sequential Multicolumn Chromatography
$\bar{t}$ (s)	mean residence time
V (mL)	Volume
v (cm/s)	Velocity
$\dot{V}$ (mL/min)	Volumetric Flow
*ε* (-)	Voidage
*σ*^2^ (s^2^)	Variance
